# A comparative Monte Carlo simulation study on shielding features of the CaF_2_–CaO–B_2_O_3_–P_2_O_5_–SrO–Ta_2_O_5_ glass system against X-ray by GEANT4 and MCNPX codes

**DOI:** 10.1038/s41598-024-64096-3

**Published:** 2024-06-12

**Authors:** Farzad Isazadeh, Akbar Abdi Saray

**Affiliations:** Department of Physics, Urmia University, Urmia, Iran

**Keywords:** Shielding, GEANT4, MCNPX, Linear attenuation coefficient, X-ray, Phy-X/PSD, Applied physics, Nuclear physics

## Abstract

Regarding to their unique physical and mechanical features, glasses and glass–ceramics are suitable materials for shielding purposes. The present study evaluates the shielding properties of the CaF_2_–CaO–B_2_O_3_–P_2_O_5_–SrO–Ta_2_O_5_ glass system using Monte Carlo GEANT4 and MCNPX codes for X-ray radiations with an energy range of 20 to 100 keV. MAC values of the Ta_0_, Ta_1_, Ta_2_, Ta_2.5_, and Ta_3_ samples of the CaF_2_–CaO–B_2_O_3_–P_2_O_5_–SrO–Ta_2_O_5_ glass were computed using Phy-X/PSD, GEANT4, and MCNPX codes and compared. According to the results, the programs have good compatibility with each other. For instance, in the energy of 40 keV and for the Ta_2_ sample, GEANT4 and MCNP codes are 1.445765406 and 1.517801204 cm^2^/g, respectively, indicating 7.419529525 and 2.829628418% differences with 1.562 cm^2^/g obtained using the Phy-X/PSD software. According to recent estimations, the Ta_3_ sample of the CaF_2_–CaO–B_2_O_3_–P_2_O_5_–SrO–Ta_2_O_5_ glass system can be selected as the best shield compared with the other samples.

## Introduction

Nowadays, with the advancement of nuclear technology in various areas of science (e.g., industries, nuclear reactors, and especially medical centers), humans, plants, and animals are exposed to neutrons, heavy particles and ions, and gamma radiation. Therefore, research on protecting humans, plants, and animals from serious hazards of those radiations has been the subject of intense research^1,2^. Over the past decades, various shielding materials, e.g., wide range of stones, concretes, alloys, polymers, and glasses have been developed and used as materials with appropriate mechanical, optical, and shielding properties for protecting from radiation dangers^3–7^. Saleh et al.^8^ studied the structure, mechanical, and nuclear shielding properties of lead-free Sn–Zn–Bi alloys. Adib estimated neutron characteristics of single-crystal magnesium fluoride^9^. Also, Elnaggar^10^ investigated the effect of heavy chemical additives on the mechanical properties of cement pastes, radiation exposure the corrosion behavior of reinforced steel upon exposure to seawater. Recently, glass and glass–ceramic production has been interesting for researchers regarding their unique physical, chemical, and mechanical features (e.g., facile production, high optical transparency, low cost, low melting point, high thermal stability, chemical persistence, and easy formatting in shapes and sizes)^11,12^. The gamma and neutron radiation features of various sorts of glasses have been studied either by applying experimental methods or using simulation codes such as Geant4, MCNPX, and FLUKA^13–15^. Alzahrani et al.^16^ evaluated the radiation shielding properties of boron silicate glasses containing CrO_3_. Moreover, Alzahrani et al.^17^ studied the optical properties and radiation shielding effectiveness of Sm^3+^‑doped Zinc Boro phosphate glasses. Saleh et al.^18^ studied the effect of the PbO on the physical, structural, and protection features of TeO_2_–B_2_O_3_ glass against gamma, neutron, proton and alpha particles. Also, Saleh explored gamma and neutron shielding features of the gadolinium silico-borate glasses system^19^. According to Madhavi et al.^20^, Ta-dopped glasses have good biocompatibility, high mechanical strength, and high coefficient of friction. Also, the Ta-dopped glass system of CaF_2_–CaO–B_2_O_3_–P_2_O_5_–SrO–Ta_2_O_5_ has in-vitro bioactivity and antibacterial properties. Moreover, the shielding properties of the glass have not been studied before. Therefore, consistent with previous studies^21–24^, in this study, the shielding parameters of the CaF_2_–CaO–B_2_O_3_–P_2_O_5_–SrO–Ta_2_O_5_ glass system are evaluated using the GEANT4 toolkit and MCNPX code for the X-ray radiations. In this respect, the bioactivity and antibacterial properties of the glass have already been studied by Madhavi^20^. The calculations are considered for X-ray photons with energy range between 20 and 100 keV. Phy-X/PSD software was selected to evaluate MAC values and to compare them with the simulation results of MCNPX and GEANT4.

## Materials and methods

### Sample details

The CaF_2_–CaO–B_2_O_3_–P_2_O_5_–SrO–Ta_2_O_5_ glass has already been synthesized by the melt quenching technique^20^. Five glass samples were prepared with stable percentages of CaF_2_, CaO, B_2_O_3_, and SrO and different chemical compositions of P_2_O_5_ and Ta_2_O_5_^20^. The samples named as Ta_0_ (0% Ta_2_O_5_), Ta_1_ (1% Ta_2_O_5_), Ta_2_ (2% Ta_2_O_5_), Ta_2.5_ (2.5% Ta_2_O_5_), and Ta_3_ (3% Ta_2_O_5_)^20^. Table 1 represents the chemical compositions and density of each sample that is simulated using MCNPX and GEANT4 codes in this study, as reported by Madhavi^20^.

**Table 1:** Chemical composition (mole %) and density details of the glass samples.

Glass	CaF_2_	CaO	B_2_O_3_	P_2_O_5_	SrO	Ta_2_O_5_	Density (g/cm^3^)
Ta_0_	10	10	10	60	10	0	2.57
Ta_1_	10	10	10	59	10	1	2.752
Ta_2_	10	10	10	58	10	2	2.83
Ta_2.5_	10	10	10	57.5	10	2.5	2.844
Ta_3_	10	10	10	57	10	3	2.903

### GEANT4 and MCNP simulation geometry

#### GEANT4 toolkit

GEANT4 toolkit is a Monte Carlo package that uses random numbers during the calculation process. GEANT4 code can be used to simulate passing various sorts of particles either charged or non-charged with any range of energy within different types of materials. Moreover, an excellent feature of this code is the flexibility of choosing arbitrary physics lists and input, and output parameters^25,26^. In the present work, GEANT4-10.7, a version of GEANT4 code is employed to simulate the set-up of the X-ray radiations and calculate the shielding parameters.

#### MCNPX code

MCNPX, an improved Monte Carlo package by Los Alamos National Laboratory, is applied for calculating the shielding features of the CaF_2_–CaO–B_2_O_3_–P_2_O_5_–SrO–Ta_2_O_5_ glass system against photons with an energy range of 20–100 keV. Also, the code is designed to simulate a wide range of energies for different particles (alpha, photon, proton, neutron, deuteron, electron, ^3^He etc.) and various types of materials (elements, compositions, and mixtures). In MCNP code, the geometry can be illustrated in 2D and 3D dimensions, which is one of the best features of the code. The geometry part of the target, source, detector, and tally key can be defined in the input file of the code^26^. In the present study, MCNPX-2.6, a version of the MCNPX code was selected to simulate the set-up of the X-ray radiations.

#### The geometry details and set-up

**Figure 1:**
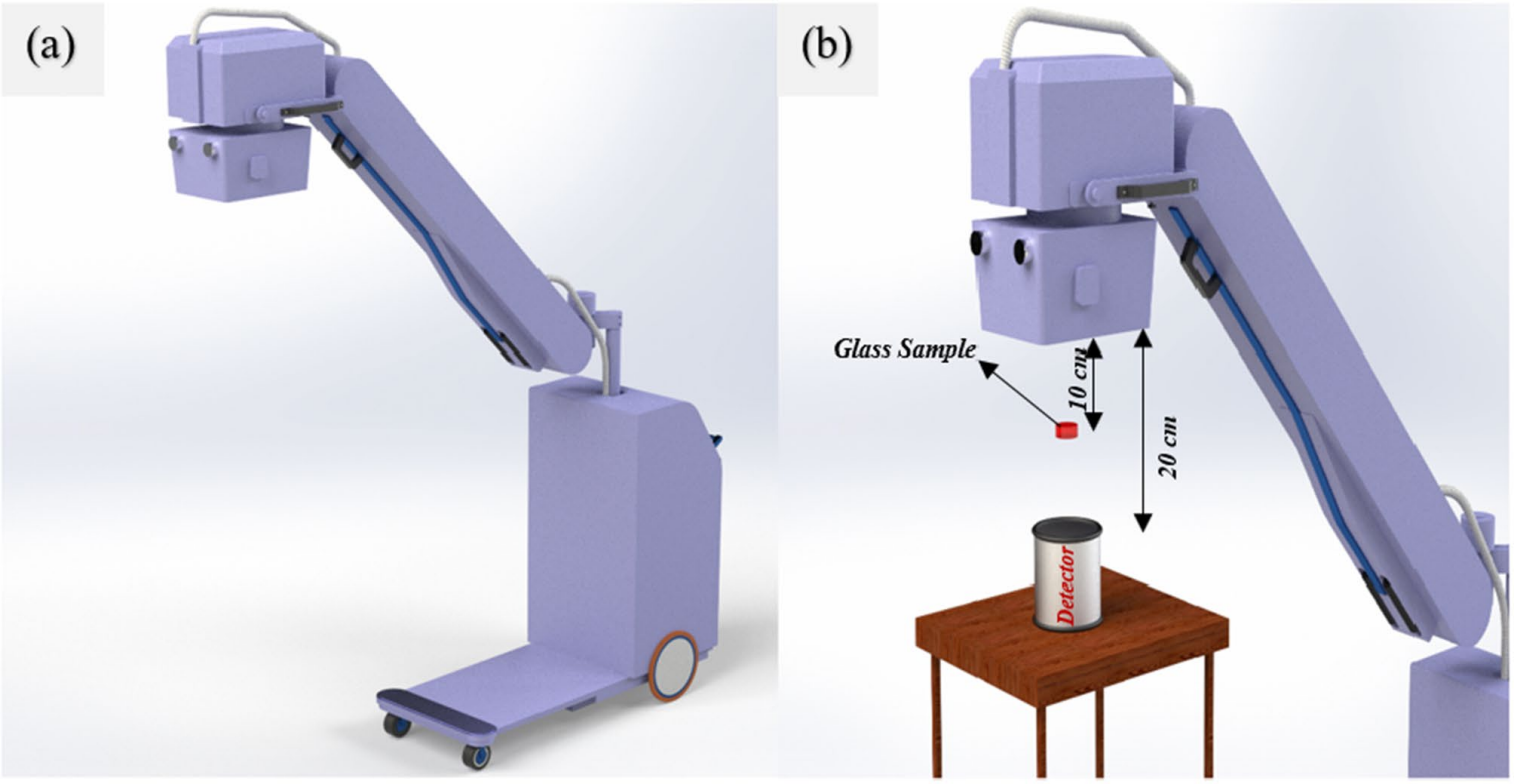
Geometry of X-ray radiation shielding simulated using MCNPX and GEANT4 codes: (**a**) before irradiating the samples, (**b**) during irradiating the sample.

Figure 1 displays the geometry of an X-ray machine, which was selected for evaluating of shielding properties of the CaF_2_–CaO–B_2_O_3_–P_2_O_5_–SrO–Ta_2_O_5_ glass system. As shown in Fig. 1, a glass sample prepared as a disk with 2 cm radius and 3 mm thickness is placed in the middle of the photon source with an energy range from 20 to 100 keV and a cylindrical detector with 2.54 cm radius and 2.54 cm thickness. The X-ray source is simulated as a circle plane with a radius of 1.5 mm, with the sample placed 10 cm far from the source and the detector. The physics lists of GEANT4 code that applied for the calculations of this study are G4EmStandardPhysics_option2, G4PhotoNuclearProcess, G4LowEGammaNuclearModel, and G4CascadeInterface.

**Figure 2:**
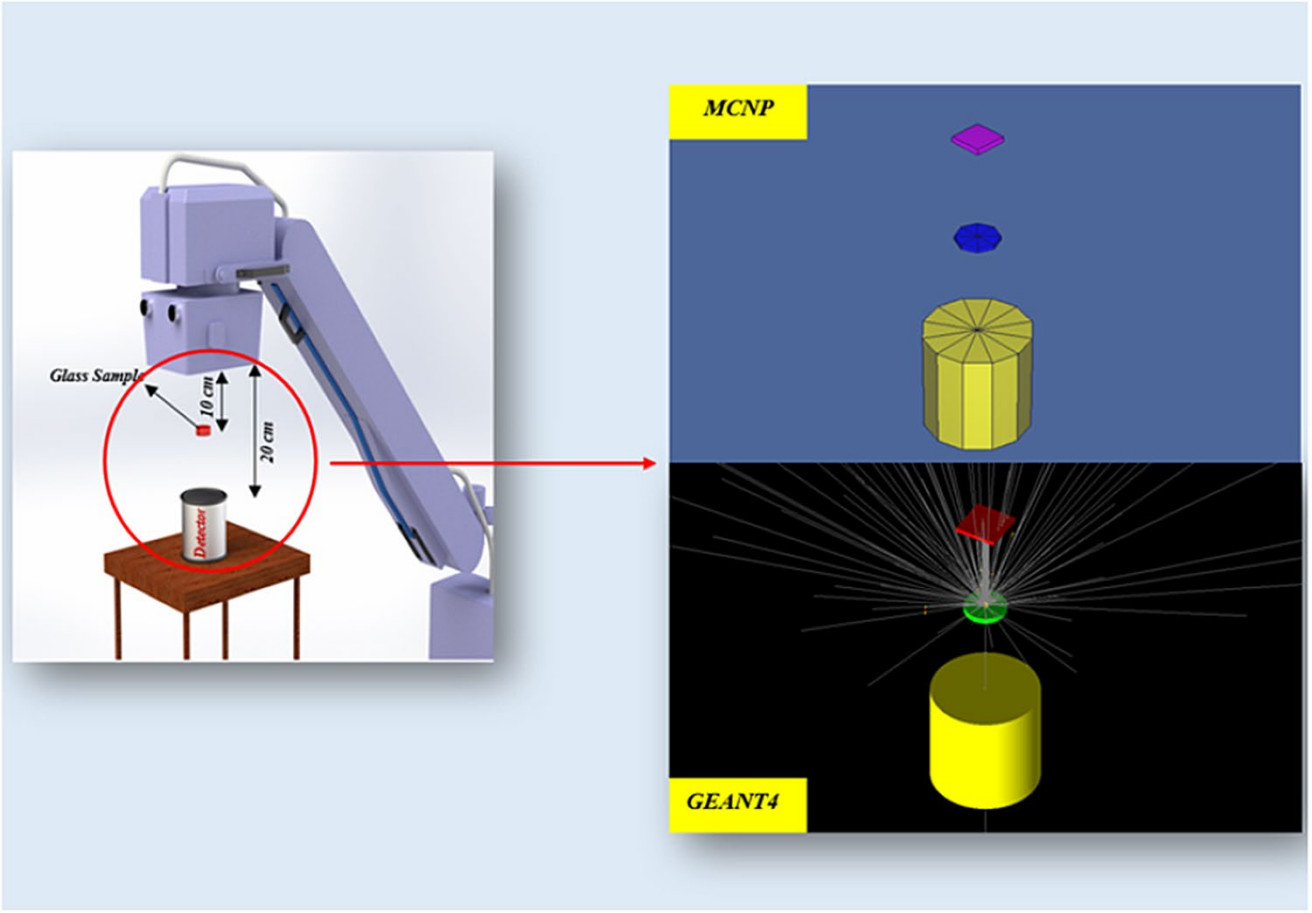
The simulation setup diagram from Visual Editor for both GEANT4 and MCNP codes.

**Figure 3:**
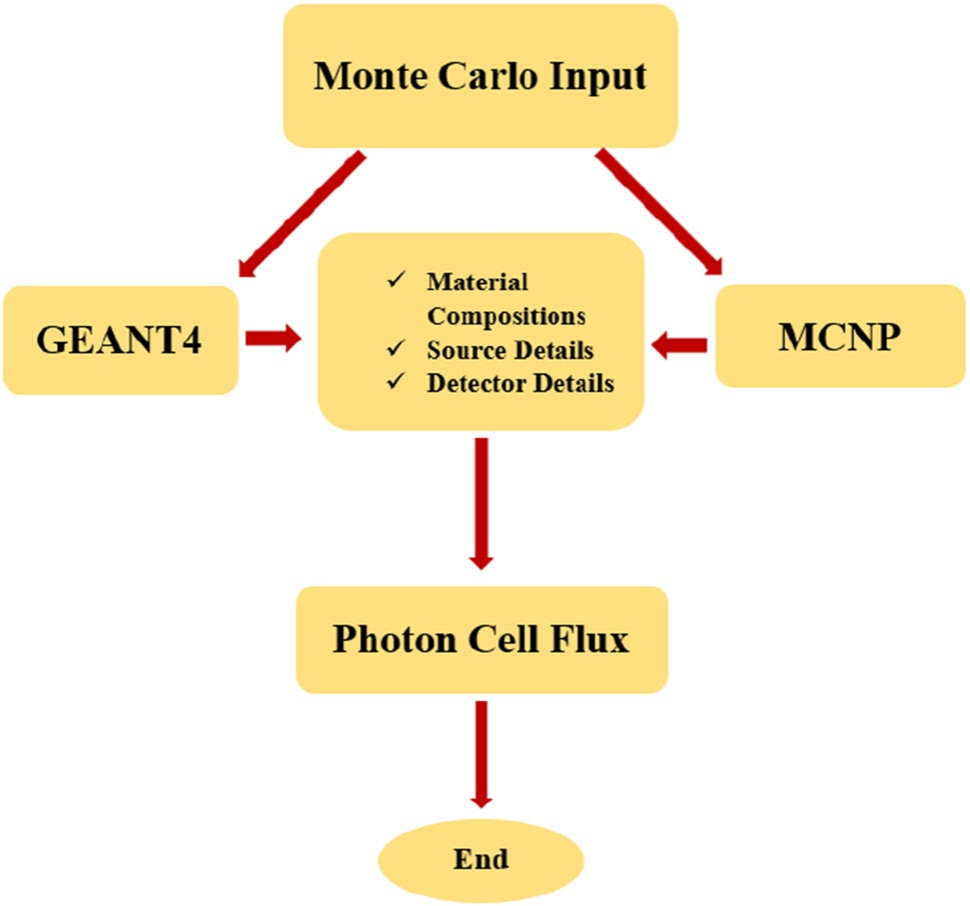
Operation of the simulation process of GEANT4 and MCNP codes.

Moreover, a simulation setup diagram from visual editor for GEANT4 and MCNP nuclear codes is displayed in Fig. 2. Photons exit from the top (the cubic), drop on the middle cylinder (the sample), and detect by the yellow cylinder (the detector). The flow chart of the overall simulation process of using GEANT4 and MCNP codes are illustrated in Fig. 3. The two Monte Carlo input files are including materials, source, and detector details, which with defining the tally, the cell flux of the photons can be seen in the output of the codes.

### Phy-X/PSD

The Phy-X/PSD software can determine shielding values for photon energies between 1 keV and 100 GeV for any materials in single elementals or mixture composites. This program illustrates the chemical compositions and density of the materials. Besides, it calculates various shielding parameters such as MAC, LAC, MFP, Z_eff_, HVL, and TVL using theoretical MAC values^27,28^. In this study, Phy-X/PSD software was selected to calculate the shielding features of the CaF_2_–CaO–B_2_O_3_–P_2_O_5_–SrO–Ta_2_O_5_ glass system and compare with it GEANT4 and MCNPX simulated values.

### Radiation parameters

#### Linear attenuation coefficient (LAC)

The linear attenuation coefficient (µ) is the number of particles or photons reflected from the beam per unit thickness of the sample surface. This coefficient is calculated in a unit of cm^−1^ using Eq. (1)^29^:

1 $$ LAC = - \frac{{\ln \left( {\frac{N}{{N_{0} }}} \right)}}{x} $$

where LAC is the linear attenuation coefficient (cm^−1^), N and N_0_ are primary and ultimate particle or photon numbers, respectively, and x is the thickness of the glass sample (cm).

#### Mass attenuation coefficient (MAC)

The mass attenuation coefficient is the probability of photons or particles that interact in a unit of mass of the glass sample. It can be computed using Eq. (2)^30^:

2 $$ MAC = \frac{LAC}{\rho } = \sum\limits_{i} {W_{i} (MAC)}_{i} $$

where LAC is the linear attenuation coefficient (cm^−1^), MAC is the mass attenuation coefficient (cm^2^/g), W_i_ is the weight fraction, and ρ denotes the glass sample density (g/cm^3^).

#### Half-value layer (HVL)

The thickness of the glass sample that cut the power of the particle or photon beam in half is defined as the half-value layer. Equation (3) is used to evaluate HVL^31^:

3 $$ HVL = \frac{\ln (2)}{{LAC}} $$

where HVL is the half-value layer (cm) and LAC is the linear attenuation coefficient (cm^−1^).

#### Tenth-value layer (TVL)

The tenth-value layer of the sample glass is the thickness of the glass that attenuates the intensity of the beam to one-tenth of its primary strength. The TVL is calculated by Eq. (4)^31^:

4 $$ TVL = \frac{\ln (10)}{{LAC}} $$

where TVL is the tenth-value layer (cm) and the LAC is the linear attenuation coefficient (cm^−1^).

#### Mean free path (MFP)

The distance between two successive interactions of the particles or photons through the glass sample is defined as the mean free path, which can be estimated by Eq. (5)^31^:

5 $$ MFP = \frac{1}{LAC} $$

where MFP is the mean free path (cm) and LAC denotes the linear attenuation coefficient (cm^−1^).

#### Effective atomic number

The effective atomic number (Z_eff_) of the samples is expressed by Eq. (6)^32^:

6 $$ Z_{eff} = \frac{{\sum\nolimits_{i} {f_{i} A_{i} \left( {MAC} \right)_{i} } }}{{\sum\limits_{j} {\frac{{A_{j} }}{{Z_{j} }}\left( {MAC} \right)_{i} } }} $$

MAC values of the GEANT4 code and Phys-x software were compared using Eq. (7)^32^:

7 $$ Diff = \left| {\frac{{MAC_{Phy - x} - MAC_{GEANT4orMCNP} }}{{MAC_{Phy - x} }}} \right| \times 100 $$

## Results and discussion

**Figure 4:**
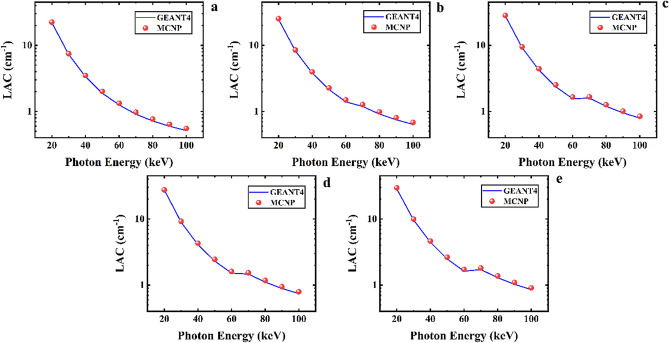
Linear attenuation coefficient values calculated using MCNPX and GEANT4 codes for (**a**) Ta_0_, (**b**) Ta_1_, (**c**) Ta_2_, (**d**) Ta_2.5_, and (**e**) Ta_3_ samples of the CaF_2_–CaO–B_2_O_3_–P_2_O_5_–SrO–Ta_2_O_5_ glass.

In this research, the shielding parameters of the CaF_2_–CaO–B_2_O_3_–P_2_O_5_–SrO–Ta_2_O_5_ glass were computed for X-ray energies from 20 to 100 keV by the GEANT4 toolkit and MCNPX code. Figure 4 indicates the linear attenuation coefficient simulated results of five samples of the CaF_2_–CaO–B_2_O_3_–P_2_O_5_–SrO–Ta_2_O_5_ glass. According to Fig. 4a, LAC values at 20 keV are 22.07342 and 22.42434066 cm^−1^ for GEANT4 and MCNP codes, respectively, while decline to 0.514242 and 0.553058358 cm^−1^ in the energy of 100 keV. Moreover, by increasing the percentages of Ta_2_O_5_ in samples, the values of LAC rise for the Ta_0_, Ta_1_, Ta_2_, Ta_2.5_, and Ta_3_ glass samples. For example, in the energy of 40 keV, the GEANT4 value of LAC reported in this study for the mentioned samples are 3.348961, 3.816376, 4.091516, 4.223683, and 4.444864 cm^−1^, while they are 3.503685242, 3.963567196, 4.295377408, 4.424974352, and 4.630099796 cm^−1^ for MCNP code, respectively. The measured values for other energies are summarized in Table 2. Due to the higher atomic number of Ta than P, with increasing the amount of Ta_2_O_5_, the density of the samples rises from 2.57 to 2.903 g/cm^3^ for the Ta_0_, Ta_1_, Ta_2_, Ta_2,5_, and Ta_3_ samples. As can be seen from Table 2, increasing the density has raised the value of the LAC parameter. Ta_3_ with the highest density value compared to other samples is the best shield against incident photons. Therefore, the behavior of the LAC is depended on the density and chemical composition of the samples, and the energy of photons.

**Table 2:** GEANT4 and MCNPX simulated values of LAC for the samples of the CaF_2_–CaO–B_2_O_3_–P_2_O_5_–SrO–Ta_2_O_5_ glass.

Photon energy (keV)	LAC simulated values (cm^−1^)					
	Ta_0_		Ta_1_			
	GEANT4	MCNP	GEANT4	MCNP		
20	22.07341833	22.42434066	24.73228459	25.33936631		
30	7.283169342	7.480468503	8.23694378	8.483611604		
40	3.348961453	3.503685242	3.816375874	3.963567196		
50	1.888805555	2.010902148	2.142324679	2.270125654		
60	1.255122077	1.33062542	1.396430247	1.494221831		
70	0.90736748	0.972357686	1.189962776	1.270451968		
80	0.717601042	0.765944149	0.91218074	0.982095674		
90	0.596790728	0.63831439	0.747960022	0.801392582		
100	0.514242323	0.553058358	0.636833196	0.681597894		

Photon energy (keV)	LAC simulated values (cm^−1^)					
	Ta_2_		Ta_2.5_		Ta_3_	
	GEANT4	MCNP	GEANT4	MCNP	GEANT4	MCNP
20	27.59448999	27.54392625	27.87242868	28.38138345	28.50927947	29.61299889
30	8.936872628	9.204437949	9.218327218	9.497209362	9.648996232	9.936976289
40	4.091516098	4.295377408	4.22368276	4.424974352	4.444864221	4.630099796
50	2.306177208	2.454236113	2.364221206	2.526734359	2.484912764	2.640002814
60	1.516947752	1.608972239	1.56307559	1.653970622	1.620872715	1.724932673
70	1.46609865	1.53717162	1.58762984	1.662693435	1.716613042	1.814044526
80	1.109765413	1.175934565	1.195950228	1.263946671	1.293085029	1.374044541
90	0.886276147	0.944419782	0.951415332	1.011259897	1.029032914	1.094886603
100	0.743778411	0.792821657	0.793705885	0.8422538	0.852066809	0.905962664

**Figure 5:**
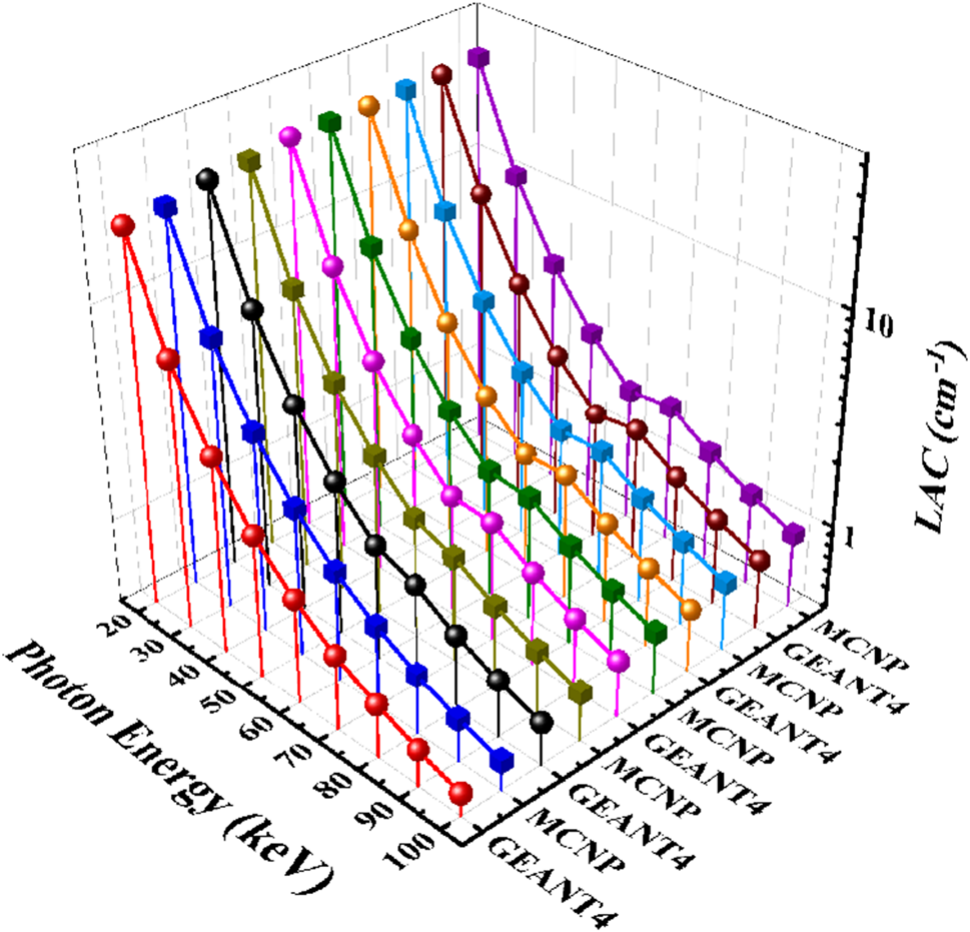
A comparison of linear attenuation coefficient parameter of the five samples of the CaF_2_–CaO–B_2_O_3_–P_2_O_5_–SrO–Ta_2_O_5_ glass that evaluated by MCNPX and GEANT4 codes.

Furthermore, Fig. 5 presents a 3D comparison of LAC results between the five samples of the CaF_2_–CaO–B_2_O_3_–P_2_O_5_–SrO–Ta_2_O_5_ glass system.

**Figure 6:**
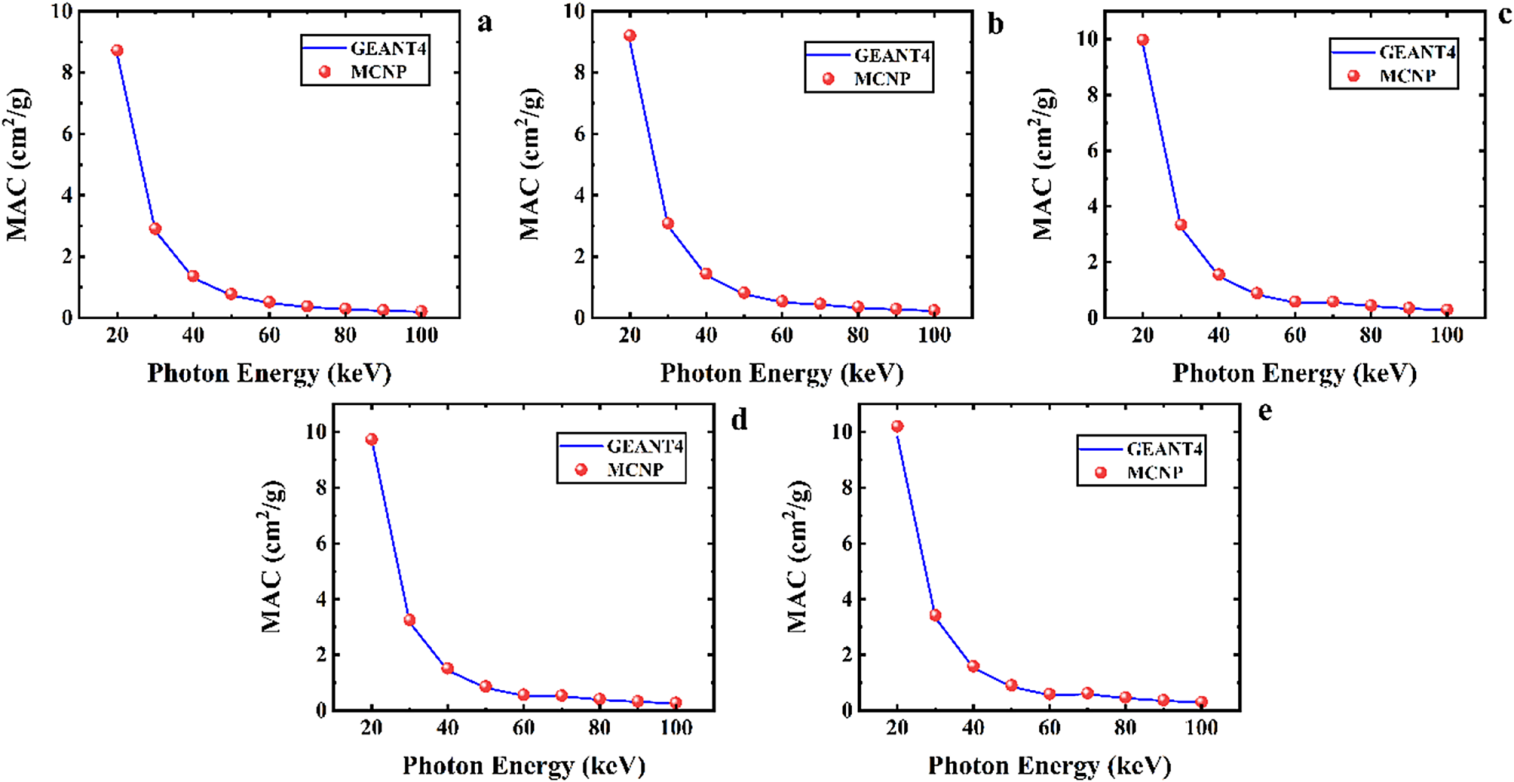
Mass attenuation coefficient values calculated using MCNPX and GEANT4 codes for (**a**) Ta_0_, (**b**) Ta_1_, (**c**) Ta_2_, (**d**) Ta_2.5_, and (**e**) Ta_3_ samples of the CaF_2_–CaO–B_2_O_3_–P_2_O_5_–SrO–Ta_2_O_5_ glass.

Figure 6. exhibits mass attenuation coefficient values estimated by MCNPX and GEANT4 codes for the Ta_0_, Ta_1_, Ta_2_, Ta_2,5_, and Ta_3_ samples of the CaF_2_–CaO–B_2_O_3_–P_2_O_5_–SrO–Ta_2_O_5_ glass. According to this figure, MAC values for the mentioned samples decline continuously from 20 to 100 keV. The values of MAC for the GEANT4 toolkit in energy of 40 keV for the Ta_0_, Ta_1_, Ta_2_, Ta_2,5_, and Ta_3_ samples are 1.303098, 1.386764, 1.445765, 1.485121, and 1.531128 cm^2^/g, respectively. Also, the simulation outputs by MAC for MCNP code for the glass samples were reported to be 1.363301651, 1.440249708, 1.517801204, 1.555898155, and 1.594936203 cm^2^/g, respectively, which are in good agreement with the GEANT4 values. Table 3 lists the values of MAC for five mentioned samples obtained using GEANT4 and MCNP codes and Phy-X/PSD program in the energy range of 20 to 100 keV. Table 3 presents the differences between GEANT4 and MCNPX codes, and the Phy-X/PSD program. As can be seen, the outputs of these three software are in good agreement for all energy range and the five samples of the glass system.

**Table 3:** GEANT4 and MCNPX simulated values of MAC for the samples of the CaF_2_–CaO–B_2_O_3_–P_2_O_5_–SrO–Ta_2_O_5_ glass.

Photon energy (keV)	MAC simulated values (cm^2^/g)								
	Ta_0_		Ta_1_			Ta_2_			
	GEANT4 MCNP	Phy-X/PSD	Diff (%)	GEANT4 MCNP	Phy-X/PSD	Diff (%)	GEANT4 MCNP	Phy-X/PSD	Diff (%)
20	8.588878726 8.725424382	8.861	3.067792953 1.530026162	8.987022017 9.207618573	9.353	3.910706042 1.554382837	9.750703176 9.73283613	9.845	0.956373472 1.139297821
30	2.833918032 2.910688133	2.968	4.515804851 1.930992808	2.993075501 3.082707705	3.138	4.624875965 1.76202342	3.157905522 3.252451572	3.308	4.551265774 1.679214872
40	1.303097842 1.363301651	1.403	7.101175471 2.829533105	1.386764489 1.440249708	1.482	6.436803927 2.817158714	1.445765406 1.517801204	1.562	7.419529525 2.829628418
50	0.734943796 0.782452198	0.815	9.789812825 3.993595365	0.778461003 0.82490031	0.859	9.33033544 3.969696103	0.814903607 0.867221241	0.902	9.699473683 3.855738202
60	0.488374349 0.517753082	0.544	10.28402044 4.824801123	0.507423781 0.542958514	0.571	11.18523018 4.910943204	0.536023941 0.568541427	0.598	10.40887461 4.926182765
70	0.353061276 0.378349294	–	–	0.432399264 0.461646791	–	–	0.51805606 0.543170184	–	–
80	0.279222195 0.298032743	0.319	12.53793957 6.57280786	0.331461025 0.356866161	0.380	12.71153351 6.087852426	0.392143256 0.415524581	0.440	10.91939508 5.562595182
90	0.232214291 0.248371358	–	–	0.271787799 0.2912037	–	–	0.313171783 0.333717237	–	–
100	0.200094289 0.215197805	0.233	14.02361459 7.640427223	0.231407411 0.247673653	0.267	13.18218857 7.238332099	0.262819226 0.280148995	0.300	12.49734808 6.61700159

Photon energy (keV)	MAC simulated values (cm^2^/g)								
	Ta_2.5_	Ta_3_							
	GEANT4 MCNP	Phy-X/PSD	Diff (%)	GEANT4 MCNP	Phy-X/PSD	Diff (%)			
20	9.800432027 9.979389399	10.091	2.87845871 1.106041039	9.820626756 10.20082635	10.337	4.994760393 1.317342078			
30	3.24132462 3.339384445	3.394	4.487643127 1.609179582	3.323801664 3.423002511	3.479	4.454214706 1.609585778			
40	1.48512052 1.555898155	1.601	7.258926849 2.817104638	1.531127875 1.594936203	1.641	6.700746492 2.807056518			
50	0.831301408 0.888443867	0.924	10.06817439 3.848066313	0.855980973 0.909405034	0.946	9.544612201 3.868389613			
60	0.549604638 0.581564916	0.612	10.16395471 4.973052875	0.558344029 0.594189691	0.625	10.7038714 4.929649447			
70	0.55823834 0.584632009	–	–	0.591323817 0.624886161	–	–			
80	0.420516958 0.444425693	0.470	10.61431927 5.441341891	0.445430599 0.473318822	0.501	11.03714977 5.525185175			
90	0.334534223 0.355576616	–	–	0.354472241 0.377156942	–	–			
100	0.279080832 0.296151125	0.317	12.03451336 6.576932131	0.293512507 0.312078079	0.334	12.16611769 6.563449287			

**Figure 7:**
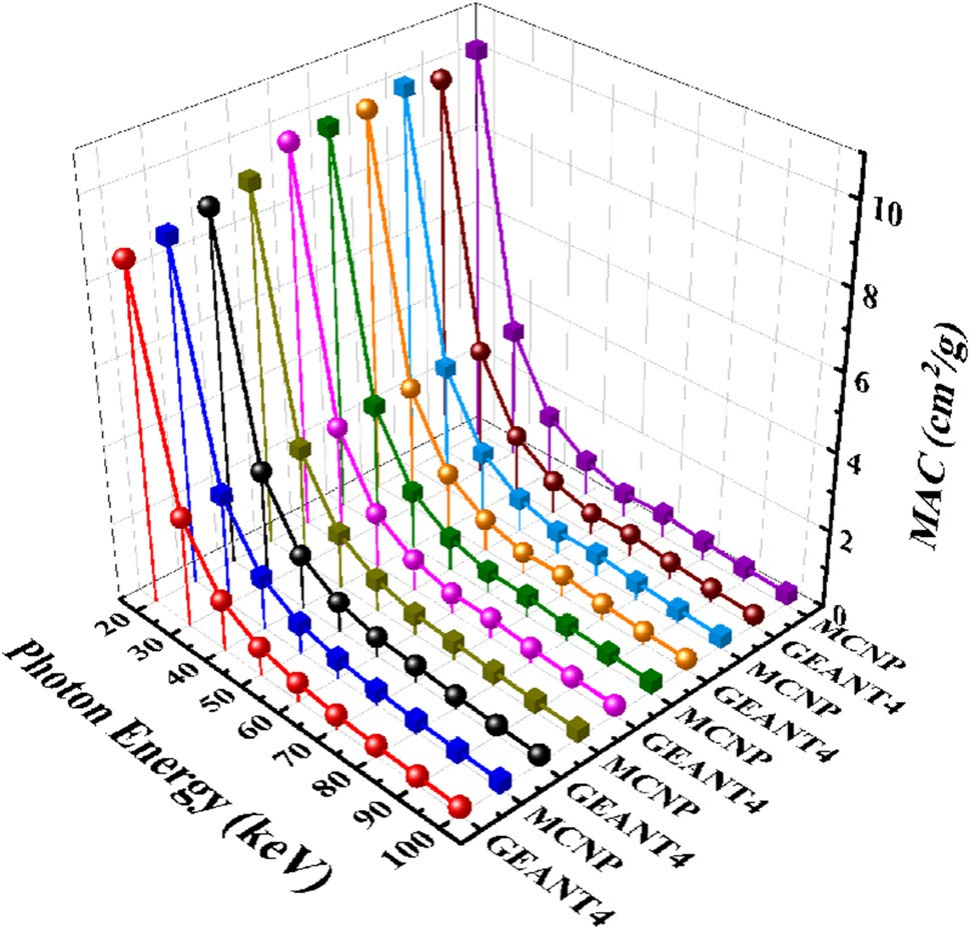
A comparison of mass attenuation coefficient parameter of the five samples of the CaF_2_–CaO–B_2_O_3_–P_2_O_5_–SrO–Ta_2_O_5_ glass that evaluated by GEANT4 and MCNPX codes.

Figure 7 compares MAC values for the CaF_2_–CaO–B_2_O_3_–P_2_O_5_–SrO–Ta_2_O_5_ glass samples.

**Figure 8:**
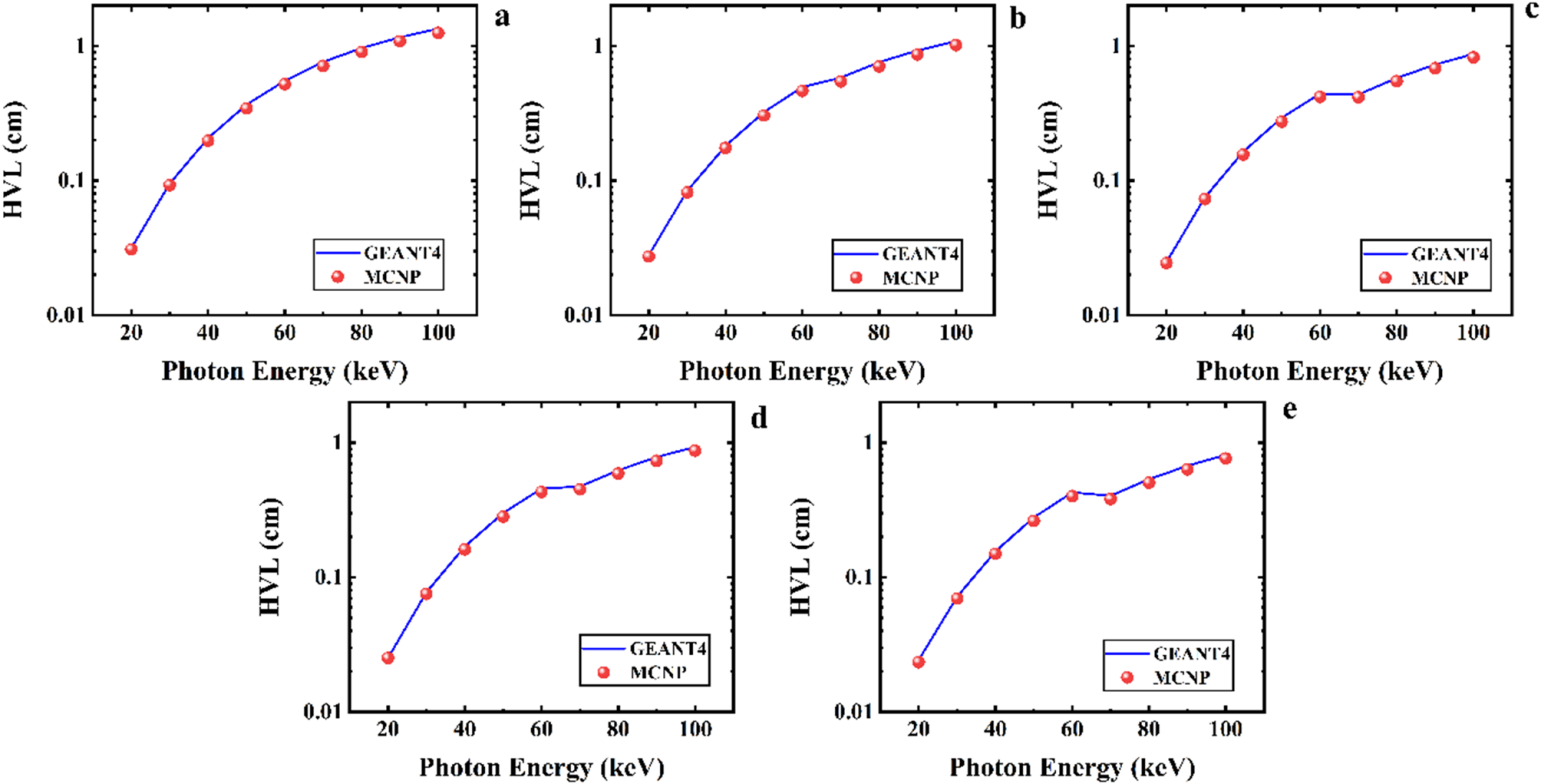
Half-value layer results calculated using GEANT4 and MCNPX codes for (**a**) Ta_0_, (**b**) Ta_1_, (**c**) Ta_2_, (**d**) Ta_2.5_, and (**e**) Ta_3_ samples of the CaF_2_–CaO–B_2_O_3_–P_2_O_5_–SrO–Ta_2_O_5_ glass.

Figure 8 presents the half-value layer GEANT4 and MCNP codes calculations of the Ta_0_, Ta_1_, Ta_2_, Ta_2,5_, and Ta_3_ samples for photons with energy ranging from 20 to 100 keV. According to this figure, HVL results for each sample start at 20 keV and gently rise to 100 keV. However, the values for samples with a higher of Ta_2_O_5_ content are less than others. For instance, in incident photons with energy of 40 keV, HVL of the five samples are obtained as 0.20693, 0.181586, 0.169375, 0.164075, and 0.15591 cm for the GEANT4 program, respectively. Meanwhile, the MCNP values were obtained as 0.19779174, 0.174842501, 0.161336231, 0.156611077, and 0.1496728 cm, respectively. Table 4 presents the HVL results for other energies of photons.

**Table 4:** GEANT4 and MCNPX simulated values of HVL for the samples of the CaF_2_–CaO–B_2_O_3_–P_2_O_5_–SrO–Ta_2_O_5_ glass.

Photon energy (keV)	HVL simulated values (cm)					
	Ta_0_		Ta_1_			
	GEANT4	MCNP	GEANT4	MCNP		
20	0.031395228	0.030903919	0.028020056	0.02734875		
30	0.095150884	0.092641256	0.084133147	0.081686908		
40	0.206929823	0.19779174	0.181585887	0.174842501		
50	0.36689854	0.344621443	0.323480379	0.305269446		
60	0.552137527	0.520807726	0.496265389	0.463786558		
70	0.763747892	0.712700696	0.582371158	0.545475168		
80	0.965717662	0.904765707	0.75971786	0.705633899		
90	1.16121107	1.085671906	0.926520107	0.864744715		
100	1.347613702	1.253032325	1.088197042	1.016728494		

Photon energy (keV)	HVL simulated values (cm)					
	Ta_2_		Ta_2.5_		Ta_3_	
	GEANT4	MCNP	GEANT4	MCNP	GEANT4	MCNP
20	0.025113709	0.025159812	0.02486328	0.024417414	0.024307875	0.023401885
30	0.077543905	0.075289768	0.075176329	0.072968803	0.071820942	0.069739524
40	0.169374868	0.161336231	0.164074823	0.156611077	0.155910274	0.1496728
50	0.300497289	0.28236892	0.29311978	0.274267058	0.27888303	0.26249972
60	0.45683841	0.430709731	0.443356677	0.418991723	0.427547452	0.401754811
70	0.472683063	0.450827996	0.436499732	0.416793611	0.403701931	0.382019289
80	0.624456297	0.589318505	0.579455552	0.548282626	0.535927634	0.504350463
90	0.781923334	0.733783867	0.728388514	0.685283775	0.673447846	0.632942259
100	0.931729115	0.874093176	0.873119392	0.82279237	0.813316506	0.764932185

**Figure 9:**
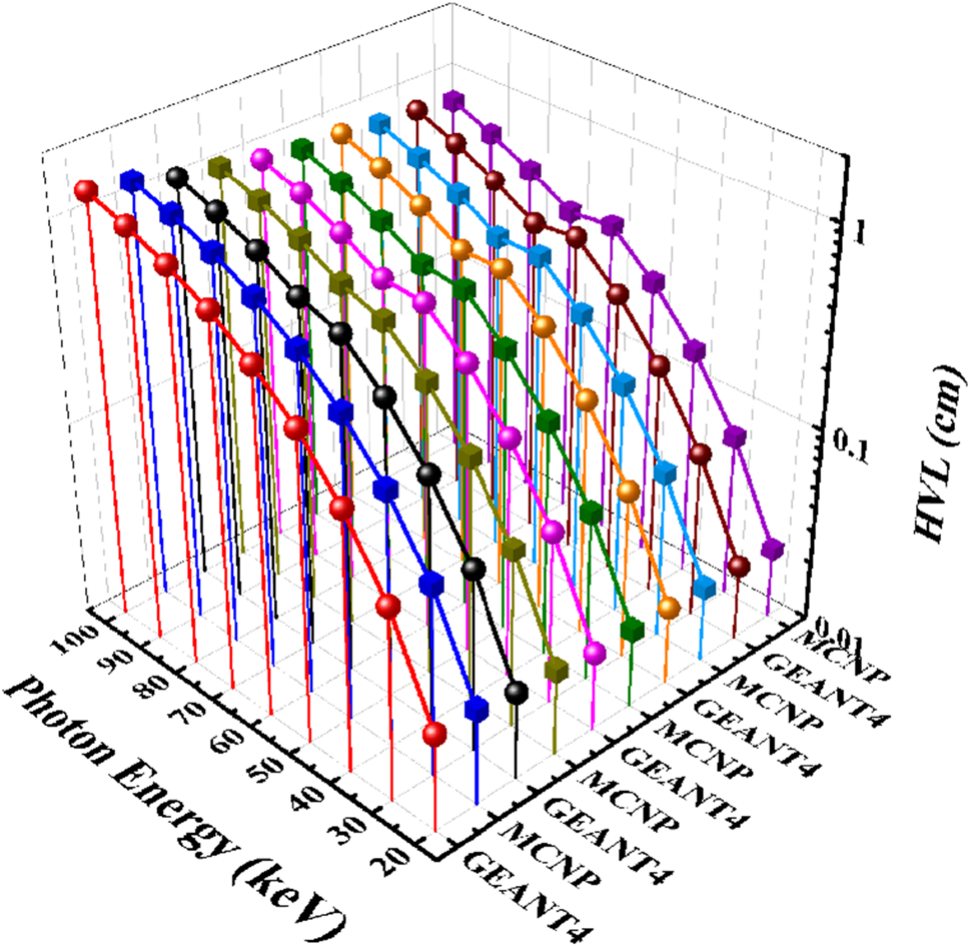
A comparison of half-value layer parameter of the five samples of the CaF_2_–CaO–B_2_O_3_–P_2_O_5_–SrO–Ta_2_O_5_ glass that evaluated by MCNPX and GEANT4 codes.

Figure 9 compares the HVL values of the Ta_0_, Ta_1_, Ta_2_, Ta_2,5_, and Ta_3_ samples for a photon energy range of 20 to 100 keV.

**Figure 10:**
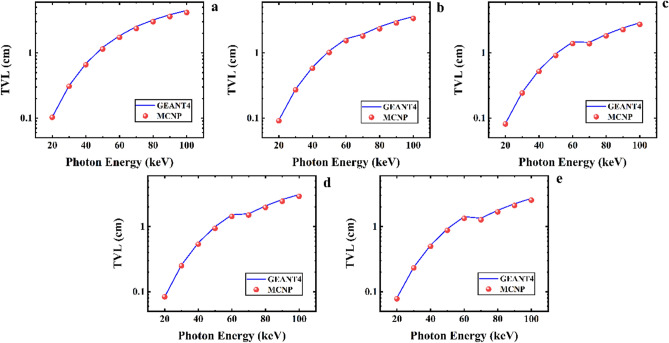
Tenth-value layer simulated measurements using GEANT4 and MCNPX codes for (**a**) Ta_0_, (**b**) Ta_1_, (**c**) Ta_2_, (**d**) Ta_2.5_, and (**e**) Ta_3_ samples of the CaF_2_–CaO–B_2_O_3_–P_2_O_5_–SrO–Ta_2_O_5_ glass.

Also, Fig. 10 presents the GEANT4 and MCNPX simulated data of the tenth-value layer for five samples of the CaF_2_–CaO–B_2_O_3_–P_2_O_5_–SrO–Ta_2_O_5_ glass irradiated by photons with an energy range of 20 to 100 keV. According to Fig. 10, in energy of 20 keV, the TVL values start and rise to 100 keV for each sample. According to GEANT4 values, in the thicknesses of 0.687552, 0.603343, 0.562771, 0.545161, and 0.518033 cm for the Ta_0_, Ta_1_, Ta_2_, Ta_2,5_, and Ta_3_ samples, the photon with an energy of 40 keV losses its power to one-tenth of its original one. Meanwhile, these values for the MCNP code are 0.657189483, 0.580937569, 0.536061183, 0.520361229, and 0.497307876 cm, respectively. The TVL values decline by increasing the amount of Ta_2_O_5_. The TVL simulated results for photon energies of 20 to 100 keV and the five samples of the glass are summarized in Table 5.

**Table 5:** GEANT4 and MCNPX simulated values of TVL for the samples of the CaF_2_–CaO–B_2_O_3_–P_2_O_5_–SrO–Ta_2_O_5_ glass.

Photon energy (keV)	TVL simulated values (cm)					
	Ta_0_		Ta_1_			
	GEANT4	MCNP	GEANT4	MCNP		
20	0.10431484	0.102682399	0.09310038	0.090869877		
30	0.316151525	0.307812952	0.279543621	0.271415666		
40	0.687551984	0.657189483	0.603343373	0.580937569		
50	1.21906942	1.14505079	1.074806781	1.014298521		
60	1.834550706	1.730453257	1.648908063	1.540992806		
70	2.537654415	2.368043288	1.935005985	1.812414126		
80	3.208725963	3.00620495	2.524264097	2.344562911		
90	3.85827893	3.607289967	3.078486851	2.873229856		
100	4.477626581	4.163367321	3.615680068	3.378216268		

Photon energy (keV)	TVL simulated values (cm)					
	Ta_2_		Ta_2.5_		Ta_3_	
	GEANT4	MCNP	GEANT4	MCNP	GEANT4	MCNP
20	0.083443655	0.083596836	0.08261157	0.081130122	0.080766162	0.07775589
30	0.257649984	0.250160315	0.249783398	0.242448598	0.238634676	0.231718888
40	0.56277063	0.536061183	0.545160521	0.520361229	0.518032718	0.497307876
50	0.998442394	0.938208464	0.973929634	0.911288947	0.926626128	0.872190393
60	1.517906658	1.431090629	1.473111798	1.39215598	1.420583536	1.334884039
70	1.570552632	1.497936251	1.450328682	1.38485246	1.341353606	1.26931013
80	2.07483948	1.958089474	1.925318494	1.821742282	1.78069117	1.675771799
90	2.598044752	2.438094941	2.420168161	2.276946905	2.23762045	2.103035225
100	3.095794473	2.904291368	2.901055838	2.733837583	2.702352761	2.541589387

**Figure 11:**
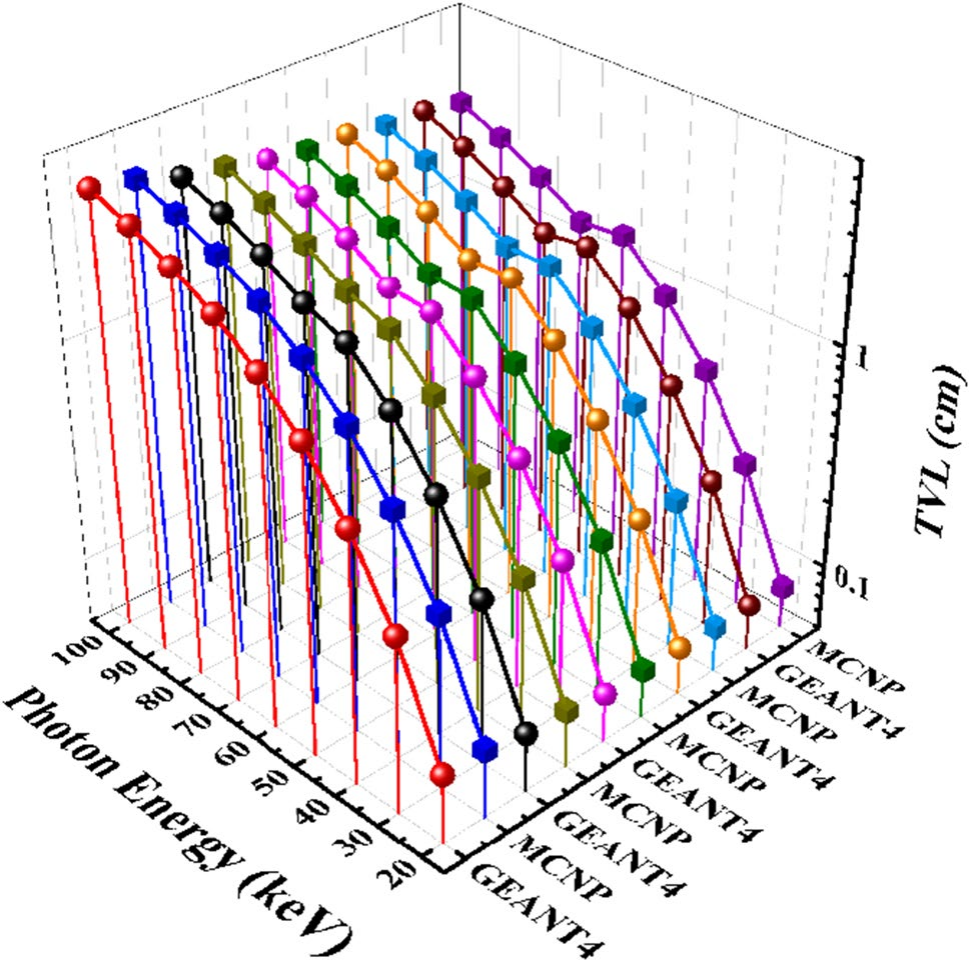
A comparison of the TVL GEANT4 and MCNPX simulated values for samples of the CaF_2_–CaO–B_2_O_3_–P_2_O_5_–SrO–Ta_2_O_5_ glass.

Moreover, Fig. 11 compares the TVL parameter values for the five mentioned samples of the CaF_2_–CaO–B_2_O_3_–P_2_O_5_–SrO–Ta_2_O_5_ glass system.

**Figure 12:**
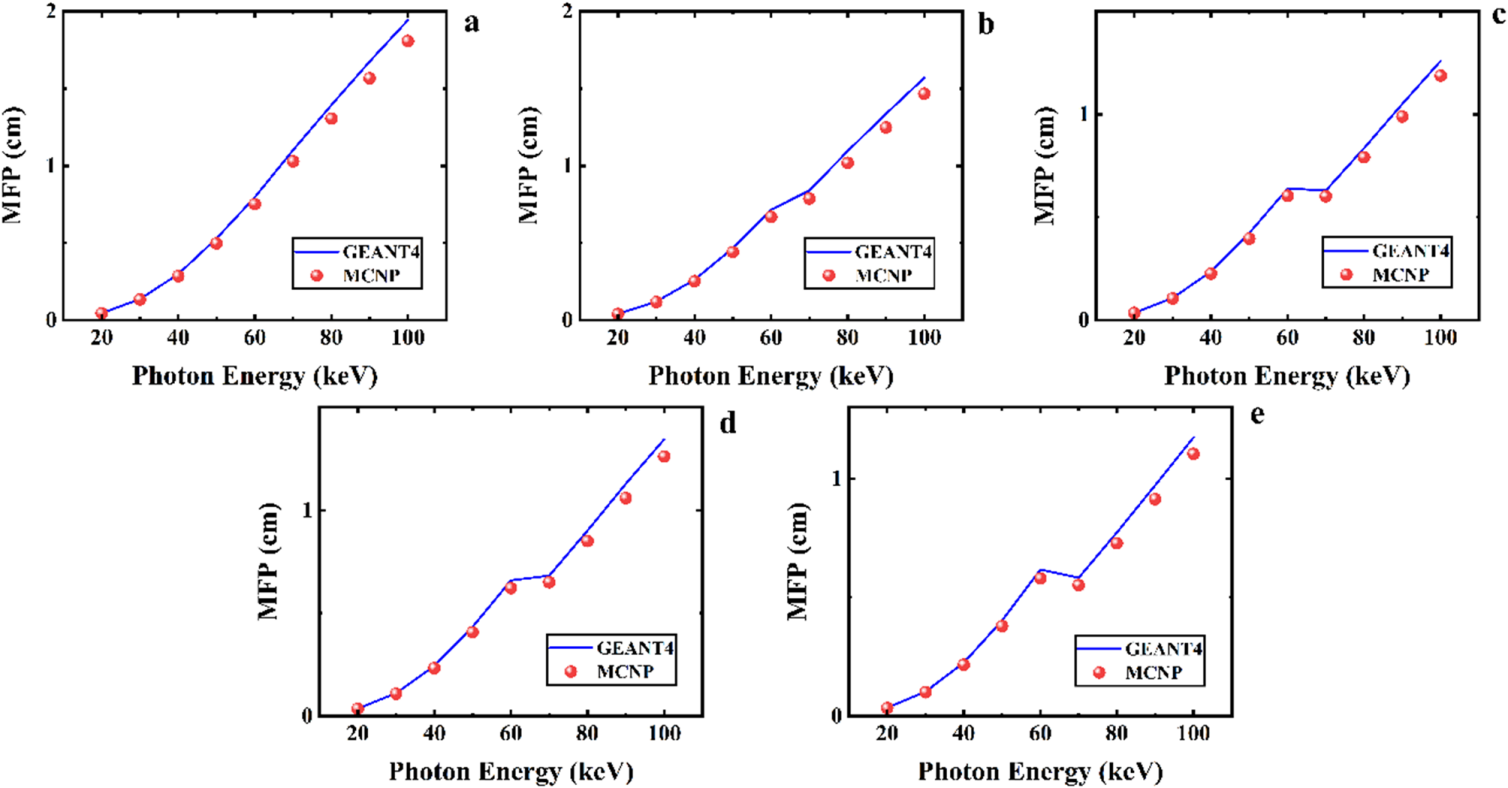
Mean free path simulated measurements using GEANT4 and MCNPX codes for (**a**) Ta_0_, (**b**) Ta_1_, (**c**) Ta_2_, (**d**) Ta_2.5_, and (**e**) Ta_3_ samples of the CaF_2_–CaO–B_2_O_3_–P_2_O_5_–SrO–Ta_2_O_5_ glass.

**Figure 13:**
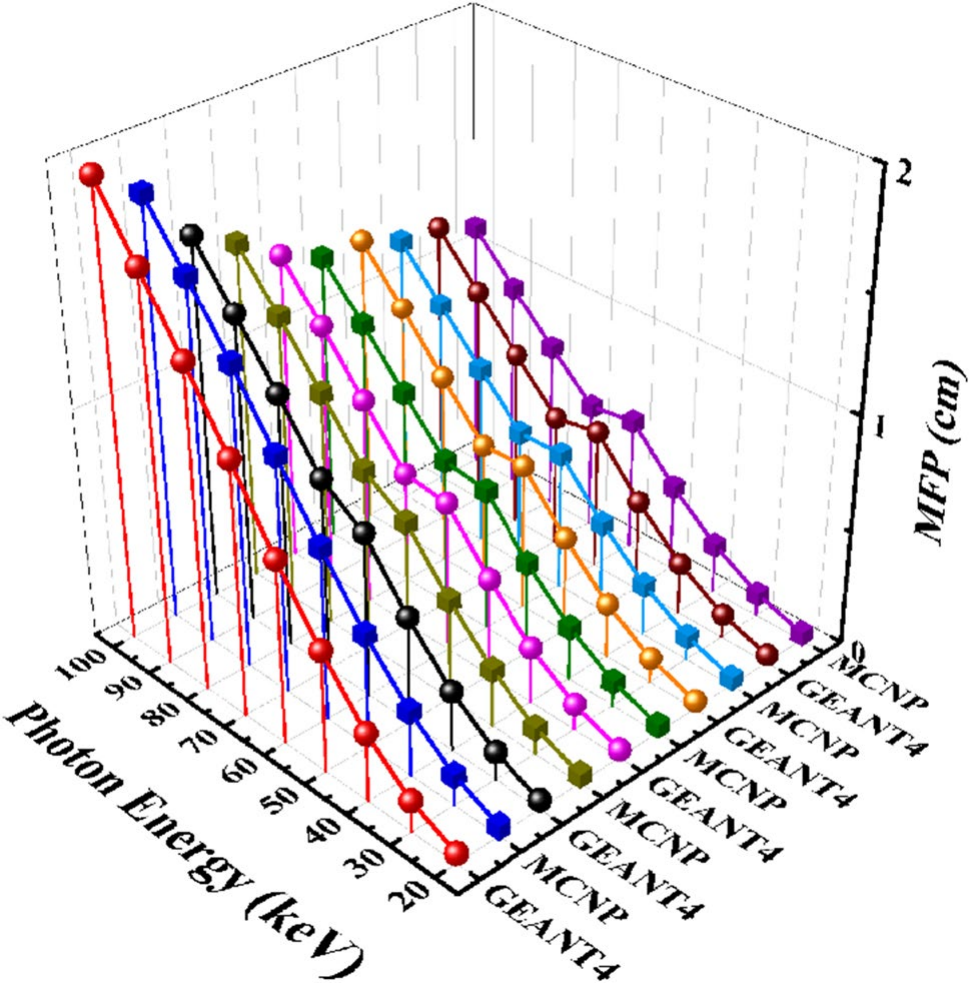
A comparison of the MFP GEANT4 and MCNPX simulated values for samples of the CaF_2_–CaO–B_2_O_3_–P_2_O_5_–SrO–Ta_2_O_5_ glass.

Figure 12 exhibits the mean free path values computed by GEANT4 and MCNPX codes for five samples of the CaF_2_–CaO–B_2_O_3_–P_2_O_5_–SrO–Ta_2_O_5_ glass system in the photon energy range between 20 and 100 keV. As can be seen, the MFP values for each sample gradually decrease from 20 to 100 keV. According to Fig. 13, the MFP values decline by increasing the Ta_2_O_5_ content of the samples. The distance between to interacting of photons with energy of 40 keV estimated by GEANT4 toolkit in the Ta_0_, Ta_1_, Ta_2_, Ta_2,5_, and Ta_3_ samples are 0.2986, 0.262029, 0.244408, 0.23676, and 0.224979 cm, and for MCNP code are 0.285413766, 0.252297981, 0.232808414, 0.22599001, and 0.215978066 cm, respectively. Table 6 presents more values of the MFP for photon energy in the range of 20 to 100 keV.

**Table 6:** GEANT4 and MCNPX simulated values of MFP for the samples of the CaF_2_–CaO–B_2_O_3_–P_2_O_5_–SrO–Ta_2_O_5_ glass.

Photon energy (keV)	MFP simulated values (cm)					
	Ta_0_		Ta_1_			
	GEANT4	MCNP	GEANT4	MCNP		
20	0.045303359	0.044594399	0.040432981	0.039464286		
30	0.137302863	0.133681467	0.121404252	0.117874326		
40	0.298600033	0.285413766	0.262028698	0.252297981		
50	0.529435122	0.497289239	0.466782654	0.440504251		
60	0.796735248	0.751526301	0.716111673	0.669244672		
70	1.10208931	1.028428133	0.840362422	0.787121454		
80	1.39353198	1.305578221	1.096273968	1.018230735		
90	1.675629249	1.566626127	1.336969852	1.247827872		
100	1.944608516	1.808127453	1.570269902	1.467140684		

Photon energy (keV)	MFP simulated values (cm)					
	Ta_2_		Ta_2.5_		Ta_3_	
	GEANT4	MCNP	GEANT4	MCNP	GEANT4	MCNP
20	0.036239119	0.036305645	0.035877749	0.035234364	0.035076299	0.033768954
30	0.111895966	0.108643244	0.108479551	0.105294088	0.103637723	0.100634234
40	0.244408179	0.232808414	0.236760206	0.22599001	0.224978751	0.215978066
50	0.433618022	0.407458759	0.422972266	0.395767761	0.402428614	0.378787475
60	0.659218486	0.621514763	0.639764325	0.60460566	0.616951591	0.579732772
70	0.682082342	0.650545448	0.629869744	0.601433781	0.582542469	0.551254385
80	0.901091337	0.850387453	0.836155198	0.79117262	0.773344349	0.727778445
90	1.1283165	1.058851179	1.051065677	0.988865476	0.971786214	0.913336594
100	1.344486457	1.261317715	1.259912542	1.187290577	1.173616892	1.103798246

**Figure 14:**
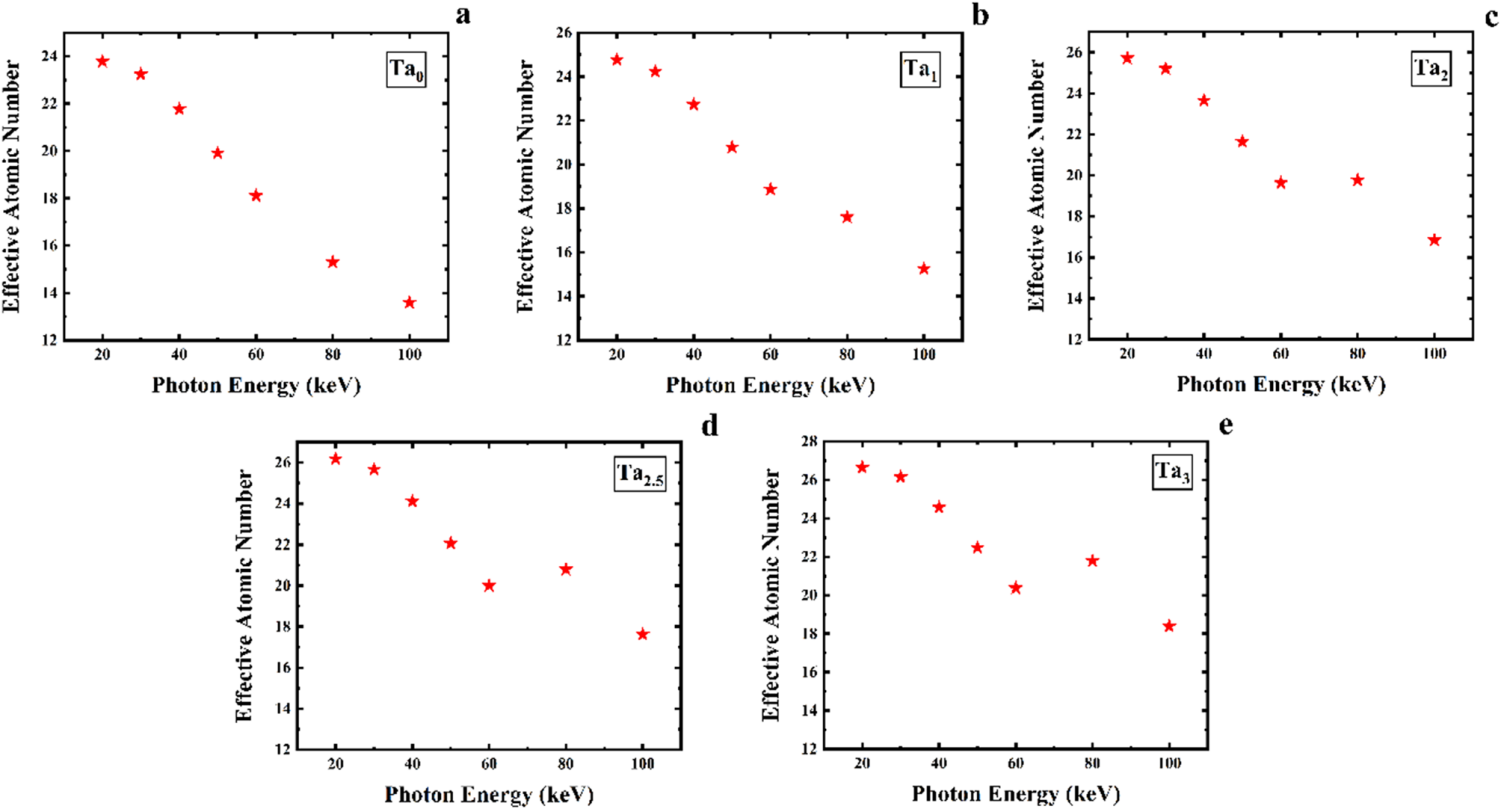
Effective atomic number for (**a**) Ta_0_, (**b**) Ta_1_, (**c**) Ta_2_, (**d**) Ta_2.5_, and (**e**) Ta_3_ samples of the CaF_2_–CaO–B_2_O_3_–P_2_O_5_–SrO–Ta_2_O_5_ glass.

**Figure 15:**
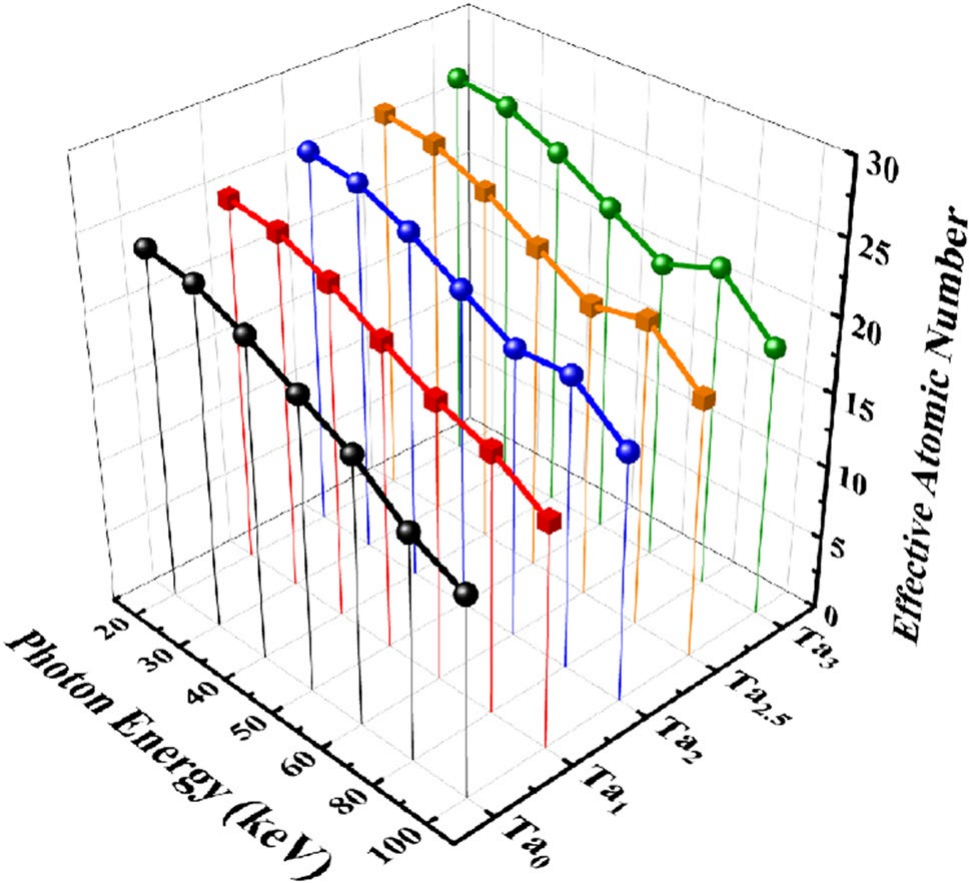
A comparison of effective atomic number for the five samples of the CaF_2_–CaO–B_2_O_3_–P_2_O_5_–SrO–Ta_2_O_5_ glass.

Figure 14 demonstrates the effective atomic number of the five samples of the CaF_2_–CaO–B_2_O_3_–P_2_O_5_–SrO–Ta_2_O_5_ glass. The values of Z_eff_ have the highest at 20 keV photon energy, which are 23.78, 24.76, 25.72, 26.18, and 26.64 for the Ta_0_, Ta_1_, Ta_2_, Ta_2.5_, and Ta_3_ samples, respectively. In this respect, the Z_eff_ values decrease with increasing photon energy and such that lowest result belongs to 100 keV energy. Also, the Ta_3_ sample with the highest density has the highest Z_eff_ value among the five samples. A comparison of Z_eff_ for the five samples of the CaF_2_–CaO–B_2_O_3_–P_2_O_5_–SrO–Ta_2_O_5_ glass system is given in Fig. 15. As can be noticed, the value of Z_eff_ rise with increasing the density of samples.

## Conclusion

This research explored the shielding properties of the CaF_2_–CaO–B_2_O_3_–P_2_O_5_–SrO–Ta_2_O_5_ glass system with five different molar fractions of Ta_2_O_5_ using GEANT4 and MCNPX nuclear codes, and PhyX/PSD program. The density of the five samples was different from 2.57 to 2.903 g/cm^3^, depending on the amount of Ta_2_O_5_. The parameter of MAC for each sample was determined using the GEANT4 and MCNP codes for photons with an energy range between 20 and 100 keV and compared with the values of Phy-X/PSD software. In the energy of 40 keV and for the sample of Ta_3_, the values of Phy-X/PSD, GEANT4, and MCNP were estimated as 1.641, 1.531127875, and 1.594936203 cm^2^/g, respectively. In contrast, the Monte Carlo codes have 6.700746492 and 2.807056518% discrepancy with the online software. Besides, the value of MAC rises by increasing the Ta_2_O_5_ content. In the mentioned energy, for Ta_0_, Ta_1_, Ta_2_, Ta_2.5_, and Ta_3_ samples, the MAC values of the Phy-X/PSD program are 1.403, 1.482, 1.562, 1.601, and 1.641 cm^2^/g. Therefore, according to the results, the Ta_3_ sample was selected as the best shield against photons in comparison with the other samples.

## Data Availability

All data generated or analyzed during this study were included in this article.
